# Association of concomitant autoimmunity with the disease features and long-term treatment and health outcomes in Celiac disease

**DOI:** 10.3389/fmed.2022.1055135

**Published:** 2022-11-16

**Authors:** Riku Tauschi, Anna Eurén, Nina Vuorela, Sara Koskimaa, Heini Huhtala, Katri Kaukinen, Laura Kivelä, Kalle Kurppa

**Affiliations:** ^1^Tampere Center for Child, Adolescent and Maternal Health Research, Faculty of Medicine and Health Technology, Tampere University, Tampere, Finland; ^2^Celiac Disease Research Center, Faculty of Medicine and Health Technology, Tampere University, Tampere, Finland; ^3^Department of Pediatrics, Tampere University Hospital, Tampere, Finland; ^4^Faculty of Social Sciences, Tampere University, Tampere, Finland; ^5^Department of Internal Medicine, Tampere University Hospital, Tampere, Finland; ^6^Children's Hospital, and Pediatric Research Center, University of Helsinki and Helsinki University Hospital, Helsinki, Finland; ^7^The University Consortium of Seinäjoki, Seinäjoki, Finland

**Keywords:** celiac disease, autoimmune disease, autoimmunity, quality of life, gastrointestinal symptoms, gluten-free diet, type 1 diabetes, thyroidal disease

## Abstract

**Background:**

Celiac disease (CeD) is often accompanied by other autoimmune diseases (AID). However, the association of co-existing autoimmunity with the presentation and treatment success in CeD is unclear. We investigated these issues with a large and well-defined cohort of Finnish patients.

**Methods:**

Adult CeD patients (*n* = 806) were collected from multiple heath care sites *via* nationwide recruitment. They were interviewed, underwent measurement of CeD autoantibodies, and filled out questionnaires to ascertain quality of life (PGWB) and gastrointestinal symptoms (GSRS) after a median of 9.7 years on a gluten-free diet. Data were supplemented retrospectively from patient records. The results were compared between CeD patients with and without a coexisting AID.

**Results:**

Altogether 185 patients had CeD+AID and 621 had CeD only. At CeD diagnosis, patients with CeD+AID were older (median 42 vs. 36 years, *p* = 0.010) and had more joint symptoms (9.1 vs. 4.2%, *p* = 0.011), whereas the groups were comparable in sex, family history of CeD, other presenting symptoms, proportion of screen-detected subjects, and severity of duodenal lesion. During follow-up on gluten-free diet, CeD+AID patients experienced poorer general health (median score 12 vs. 14, *p* < 0.001) in PGWB, more overall gastrointestinal symptoms (2.1 vs. 1.9, *p* = 0.001), and constipation (2.0 vs. 1.7, *p* < 0.001) in GSRS, whereas there was no difference in histological and serological recovery, dietary adherence, use of gluten-free oats, smoking, and presence of regular follow-up.

**Conclusions:**

Co-existing AID was not significantly associated with the baseline features or with most long-term outcomes in CeD. However, the increased prevalence of gastrointestinal symptoms and reduced poorer self-perceived health during treatment indicates these patients' need for special support.

## Introduction

Celiac disease (CeD) is an immune-mediated condition driven by ingested gluten in genetically susceptible individuals. The disease may manifest at almost any age with heterogenous gastrointestinal and extraintestinal symptoms (1). Although recognition of the multifaceted clinical presentation and introduction of sensitive and non-invasive autoantibody tests have improved case-finding, the majority of patients remain unrecognized and thus susceptible to possibly severe complications related to untreated CeD (2–4). Interestingly, CeD is known to be frequently accompanied by other autoimmune disorders (AID) (5–7) and vice versa, indicating that targeted serological testing of the AID patients could improve the suboptimal diagnostic yield.

Nevertheless, this approach remains debatable, particularly in the absence of data on the long-term prognosis of undetected CeD in AID patients. Acceptance of the treatment—strict and life-long gluten-free diet (GFD)—in these often asymptomatic individuals may also be suboptimal (8). Therefore, the current screening recommendations are inconsistent and are based primarily on the increased prevalence figures (9–12). Additional long-term data in this multifaceted group of patients with AID could help to better evaluate the advisability of screening, as well as to formulate more individualized follow-up strategies. The possible association of coexisting AID with the clinical and histological features of CeD are also of interest as this information might provide new insights into the pathogenesis and even have prognostic value (13).

There is a long tradition of active case finding and risk group screening of CeD in Finland (14–16), and systemically maintained patient databases provide reliable medical data. We exploited these advantages to study the association of a coexisting AID with baseline features and long-term outcomes in CeD.

## Materials and methods

### Patients and study design

The study was conducted at Tampere University and Tampere University Hospital. The study population was formed by nationwide recruitment of subjects with biopsy-proven CeD *via* media advertisements and with the help of the Finnish Celiac Society (17). The recruitment was open in 2006–2014. All adult participants were interviewed systematically by a study nurse or a physician. They also completed structured questionnaires to elicit gastrointestinal symptoms and quality of life, and underwent analysis of serum CeD autoantibodies. The diagnoses and all other relevant medical data were confirmed from individual patient records. Exclusion criteria were refusal to participate, current age < 18 years, CeD unconfirmed and non-response to the questionnaires. For the study analyses, the participants were divided into two main groups based on the presence (CeD + AID group) or absence (CeD group) of a co-existing AID.

### Ethical aspects

The study design and patient recruitment were approved by the Ethics Committee of Pirkanmaa Hospital District and the Declaration of Helsinki was strictly followed. All study participants provided written informed consent.

### Medical history

The following clinico-demographic data were collected from all participants: sex, age during follow-up on GFD and at CeD diagnosis, year of diagnosis, clinical presentation at diagnosis, family history of CeD, presence of possible CeD-related symptoms in childhood (< 18 years), and other medical history. The clinical presentation of CeD was further divided into either “gastrointestinal” (e.g., abdominal pain, diarrhea, constipation, and vomiting) or “extra-intestinal” (e.g., anemia, poor growth, rash, arthralgia) (18).

The possible presence of chronic co-morbidities was confirmed from the patient records. The definition of AID was based on the literature (19–21) with some modifications, aiming to accept only conditions which have been widely recognized as belonging to the family of AIDs. Diseases with self-limiting or only temporary course (e.g., Guillain-Barré syndrome) were not considered to be AIDs. Dermatitis herpetiformis (DH) was categorized as a dermatological form of CeD (22).

The results of diagnostic histology were collected from the pathology reports. In our clinical routine, at least four representative duodenal biopsies have for decades been taken upon upper gastrointestinal endoscopy. Only samples of adequate quality and with proper orientation in the initial visual inspection were accepted for further analyses (23). The severity of the mucosal lesion was further categorized into partial villous atrophy and subtotal/total villous atrophy. The presence of a possible repeat biopsy, conducted ~12 months on a GFD after diagnosis, was also recorded and the histological findings were classified as normal, partial, and subtotal/total atrophy.

The results of baseline serological measurements, including serum endomysial antibodies (EmA) or anti-reticulin antibodies and tissue transglutaminase antibodies (TGA) were also recorded when available and classified as positive or negative.

### Evaluations during GFD

Self-reported adherence to the GFD was evaluated as a part of the interview. Adherence was further categorized to strict diet (no reported lapses), occasional lapses (lapses less than once a month) and no diet (lapses more frequent than once a month) (17). The use of gluten-free oats as a part of the diet was also elicited, as were the presence of current or previous smoking, and regular follow-up for CeD.

All participants underwent measurement of CeD serology at the time of the interviews. Serum IgA class EmA were measured by indirect immunofluorescence with human umbilical cord as a substrate (24). Titers from 1:≥5 were considered a positive result and further diluted up to 1:4000. A commercial serological test (QUANTA Lite h-tTG IgA, ELISA, INOVA Diagnostics, San Diego, CA, USA) was used to measure IgA class TGA, values >30.0 U/l being considered positive (25). IgG-class EmA and TGA were measured in case of selective IgA deficiency. The results were categorized as positive and negative. Owing to the frequently slow normalization of the autoantibodies despite a strict GFD, only participants who had been on the diet >2.0 years were included in the serological analyses (26).

The validated Gastrointestinal Symptom Rating Scale (GSRS) was used to evaluate current gastrointestinal symptoms. The survey consists of 15 questions further divided into five subgroups representing indigestion, diarrhea, constipation, abdominal pain, and reflux. The questions are rated on a Likert scale from 1 to 7 and the final points are given within the same range as an average of the scores on each subgroup. Values for each sub-dimension score are calculated as a mean of the relevant items. The total GSRS score is calculated as a mean value of all 15 items and thus also has a range between 1 and 7. Higher scores indicate more severe gastrointestinal symptoms (27, 28).

Health-related wellbeing was estimated using the Psychological General Well-Being (PGWB) questionnaire, which has 22 questions rated on a Likert scale from 1 to 6, higher scores denoting better wellbeing. The questions are further classified into six groups representing anxiety, depression, wellbeing, self-control, general health, and vitality. The results are given as a sum of each individual question and may range as follows: anxiety 5–30 points, depressed mood 3–18 points, positive wellbeing 4–24 points, self-control 3–18 points, general health 3–18 points, and vitality 4–24 points (29, 30).

### Statistics

Categorical variables are reported as numbers and percentages and continuous variables as medians with quartiles. Cross-tabulation with Chi-Square or Fisher's exact test were used to test the statistical significance for categorical variables and Mann-Whitney *U*-test for continuous variables. *P*-value < 0.05 was considered significant. Binary logistic regression was used to ascertain if CeD diagnosis in childhood was significantly associated with the frequency of concomitant AIDs in the evaluation while on GFD. The possible effect of current age on self-perceived PGWB general health (31) was controlled for by dividing CeD + AID and CeD groups into four age quartiles and comparing the results. The evaluations while on GFD were also carried out separately for the most common coexisting AIDs to assess possible differences between these clinically heterogenous diseases and/or the possible major effect of a single AID. Statistical software SPSS (version 25, IBM Corporation, Armonk, NY, USA) was used for all statistical analyses.

## Results

Of the altogether 1,035 enrolled CeD patients, 229 were excluded since they were either children at the time of the present study, had unclear CeD diagnosis or did not respond to the study questionnaires. The median age of the remaining 806 subjects was 49 (interquartile range 34–60) years and 74.9% were females. Concomitant AID was present in 185 (23.0%) subjects and 621 (77.0%) had only CeD. There were 21 different AIDs in total, the most common of these being thyroid disease, type 1 diabetes (T1D), rheumatoid arthritis, Sjögren's syndrome and psoriasis (Supplementary Table S1). Median age at CeD diagnosis was between 34 and 50 years in all other patients with AIDs apart from the patients with T1D or inflammatory bowel disease (IBD). There was also a clear female preponderance in the most of AIDs except again for T1D and IBD (Supplementary Table S1).

CeD + AID patients had received CeD diagnosis at an older age and less often in childhood than those with CeD only. However, the statistical significance of the latter disappeared after adjusting for current age (Table 1), and there was no significant association between the time of CeD diagnosis in childhood or adulthood and the frequency of coexisting AIDs during follow-up on GFD (logistic regression, data not shown). The study groups did not differ in the clinical presentation of CeD at diagnosis, but in more detailed analysis, arthralgia was more common in those with CeD + AID (Table 1). The frequency was higher in CeD patients having an AID likely to cause joint symptoms (i.e., ankylosing spondylitis, IBD, psoriasis, rheumatoid arthritis, Sjögren's syndrome, systemic lupus erythematosus) than in those with other AID (2.2 vs. 4.9% respectively; *p* = 0.040). The CeD + AID and CeD only groups were comparable in sex, presence of CeD in first-degree relatives, positivity for CeD autoantibodies, degree of histological damage at diagnosis, and presence of symptoms in childhood (Table 1). Moreover, in separate analysis, CeD + T1D patients were more often (48.0%) screening-detected compared to those with thyroid disease (8.7%), rheumatoid arthritis (18.2%), Sjögren's syndrome (18.2%) and psoriasis (10%), and all patients with AID (13.0%). Patients with T1D were also significantly younger at the time of the CeD diagnosis than the other CeD + AID patients (21.3 vs. 40.3 years, *p* < 0.001).

**Table 1:** Demographic data and clinical and histological characteristics at celiac disease (CeD) diagnosis in 806 patients with or without coexisting autoimmune disease (AID).

	**CeD** + **AID** ***n*** = **185**		**CeD only** ***n*** = **621**		
	**Median**	**Quartiles**	**Median**	**Quartiles**	***P*-value**
Age at diagnosis, years	42	24, 50	36	14, 48	**0.010**
Age at follow-up, years	54	42, 62	48	31, 60	**< 0.001**
Year of diagnosis	1997	1991, 2003	2000	1992, 2004	**0.014**
	***n***	**%**	***n***	**%**	
Females	144	77.8	460	74.1	0.300
Childhood diagnosis	40	21.6	199	32.0	**0.006** ^a^
*Clinical presentation*					0.236
Gastrointestinal	115	68.0	376	63.0	
Extra-intestinal	32	18.9	149	25.2	
Screen-detected	22	13.0	67	11.3	
CeD in relative(s)^b^	92	55.1	320	59.7	0.291
*Symptoms*					
Abdominal pain	99	55.3	281	48.1	0.092
Diarrhea	78	43.6	246	41.7	0.655
Anemia	39	21.7	159	26.2	0.215
Poor growth^c^	11	31.4	87	46.8	0.094
Rash	28	15.7	79	13.6	0.464
Arthralgia	16	9.1	24	4.2	**0.011**
Constipation	14	7.9	60	10.3	0.345
Vomiting	7	3.9	35	5.9	0.295
Asymptomatic	18	9.8	71	11.7	0.480
Seropositivity^d^	68	86.1	269	88.2	0.608
*Severity of histology^e^*					0.091
Partial atrophy	60	38.2	165	31.0	
Total/subtotal atrophy	97	61.8	367	69.0	
Symptoms in childhood	47	26.0	146	23.9	0.569

^a^P = 0.944 when adjusted by age at follow-up on GFD. ^c^Only childhood diagnoses included; ^d^Reticulin-, endomysium- and transglutaminase 2 antibodies. Data was available in >90% of the subjects in each category except in ^b^703, ^d^384, ^e^689. Bolded values denote statistical significance.

After a follow-up of median 9.7 (quartiles 5.7 and 16.1) years, CeD + AID and CeD only patients did not differ in dietary compliance, use of gluten-free oat products, current or previous smoking status, frequency of CeD seropositivity and presence of regular follow-up for CeD (Table 2). In both groups the percentage of positive EmA (CeD + AID 2.4%, CeD 2.1%) and TGA (CeD + AID 6.0%, CeD 9.0%) was further decreased in patients being ≥4 years on a GFD. The groups also had comparable histological recovery on GFD (Table 2).

**Table 2:** Follow-up data after a median of 9.7 years on dietary treatment of 806 celiac disease (CeD) patients with or without coexisting autoimmune disease (AID).

	**CeD** + **AID** ***n*** = **185**		**CeD only** ***n*** = **621**		
	***n***	**%**	***n***	**%**	***P*-value**
*Gluten-free diet*					0.497
Strict diet	174	95.1	595	96.6	
Occasional lapses	8	4.4	17	2.8	
No diet	1	0.5	4	0.6	
Use of oats	150	82.9	533	87.1	0.149
Regular long-term follow-up	58	31.9	173	28.5	0.374
Current or previous smoking	56	30.6	183	29.9	0.856
*Current seropositivity^a^*					
EmA positive^b^	7	4.8	27	6.3	0.514
TGA positive^c^	13	13.3	57	8.9	0.159
*Histology in repeat biopsy after 1 year^d^*					0.775
Normal	53	57.6	142	54.8	
Partial villous atrophy	33	35.9	103	39.8	
Subtotal/total atrophy	6	6.5	14	5.4	

^a^Patients who had been on the diet < 2 years excluded. ^b^Data on 573 subjects, including 145 with CeD + AID and 428 with CeD. ^c^Data on 574 subjects, including 146 with CeD + AID and 428 with CeD. ^d^Repeat endoscopies were performed outside the study protocol a median of 12 months after the diagnosis and histological data was available from 351 subjects. EmA, endomysial antibodies; TGA, tissue transglutaminase antibodies.

When evaluated by GSRS, patients in the CeD + AID group suffered more overall gastrointestinal symptoms and constipation than those with CeD only on GFD (Table 3). Current general health was also poorer in CeD + AID than in CeD only patients, while there was no significant difference between the groups in PGWB total or other sub-scores (Table 3). These results were not significantly affected by current age (data not shown). Complete questionnaire data were available from >90% of the participants.

**Table 3:** Psychological General Well-being (PGWB) and Gastrointestinal Symptom Rating Scale (GSRS) scores of GFD treated celiac disease (CeD) patients with or without coexisting autoimmune disease (AID).

	**CeD** +**AID** ***n*** = **185**		**CeD only** ***n*** = **621**		
	**Median**	**Quartiles**	**Median**	**Quartiles**	***P*-value**
*PGWB^a^ scores*					
Total	103	91, 113	106	95, 115	0.060
Positive wellbeing	17	15, 19	18	15, 20	0.078
Vitality	18	15, 20	18	16, 20	0.538
General health	12	10, 14	14	11, 16	**< 0.001**
Self-control	16	13, 17	16	14, 17	0.242
Depressive mood	16	15, 18	17	15, 18	0.976
Anxiety	24	21, 27	24	21, 27	0.953
*GSRS^b^ scores*					
Total	2.1	1.6, 2.9	1.9	1.5, 2.4	**0.001**
Diarrhea	1.3	1.0, 2.3	1.3	1.0, 2.0	0.666
Indigestion	2.5	1.8, 3.3	2.3	1.8, 3.0	0.078
Constipation	2.0	1.3, 3.0	1.7	1.0, 2.3	**< 0.001**
Pain	2.0	1.3, 2.7	2.0	1.3, 2.3	0.096
Reflux	1.5	1.0, 2.0	1.0	1.0, 2.0	0.094

Higher scores denote either ^a^Better self-perceived wellbeing or ^b^More severe gastrointestinal symptoms. Data was available >90% of subjects with each item. Bolded values denote statistical significance.

In additional separate analysis of the two most prevalent coexisting AIDs, PGWB general health median score was 12 (quartiles 10, 14) in subjects with thyroid disease and 13 (quartiles 10, 14) in those with T1D, compared to 12 (quartiles 10, 14) in all patients with AID. The corresponding figures for GSRS total score were 2.0 (quartiles 1.3, 3.0) in thyroid disease, 2.0 (quartiles 1.3, 2.8) in T1D and 2.1 (quartiles 1.6, 2.9) in all AID patients.

## Discussion

We found coexisting AID in 23% of the CeD patients, which is close to the figure of 21.8% observed in a previous Finnish study (32). For comparison, prevalences of 27–35% have been reported from other countries (21, 33, 34), while those in general population have been 3–5% (19, 35). Possible reasons for the somewhat variable percentages might be differences in the study designs, age and gender of the patients and ethnic variation (36, 37), as well as the challenging and often inconsistent definition of AID (35). Moreover, we used strict criteria and ruled out temporary and debatable conditions (20), which might have led to lower prevalences compared to other studies. For example, DH has often been classified as a separate AID (34, 38), whereas we, like some other authors (39, 40) consider it an extraintestinal manifestation of CeD (1, 41). One more factor possibly affecting to the country differences is variation in the diagnostic yield of CeD, this being exceptionally high in Finland (2). Regardless of the definitions, both here and in earlier studies the most common co-existing AIDs were thyroid disease and T1D (20, 33). The here observed T1D prevalence of 3.3% is close to our previous finding (42). While there are many studies on the prevalence of CeD in T1D, research on the other way round is very limited. A Swedish study reported a figure of 1.0%, but only subjects aged < 20 years were included (43), and more studies are called for.

CeD + AID patients were older at CeD diagnosis than those with CeD only, which may be attributed to several factors. Although the screening of CeD in AID patients should increase diagnostic yield, the long time needed for many AIDs to develop during the life course (44) may actually delay the first screening for CeD. Conversely, with case-finding the symptoms of CeD may be erroneously attributed to relapsing AID. Accordingly, arthralgia could have been a symptom of both untreated CeD and active AID. Of note, co-existing AID was not associated with delayed CeD diagnosis in our earlier study, but only T1D and thyroid diseases were analyzed (45). Somewhat surprisingly, the CeD + AID and CeD groups nevertheless did not differ in the proportion of screen-detected CeD, of which the overall percentage was also quite low (46, 47). This likely reflects the fact that systematic screening of CeD was recommended mainly to patients with T1D (48% screen-detected) in the era when most of the study patients were diagnosed (48). It has been debated whether early detection of CeD could prevent other AIDs (32, 49), but according to our adjusted results at least childhood diagnosis showed no protective association. However, the study was not specifically designed to address this question.

In addition to the similar proportion of screen-detected patients, CeD + AID and CeD only groups were comparable at diagnosis as regards main clinical presentation and frequency of distinct symptoms except arthralgia, as well as seropositivity for CeD and severity of histological damage. According to these results, a co-existing AID does not seem to be directly associated with the presentation of untreated CeD, but instead more indirectly, depending on whether the patients are found at early disease stage in AID-based risk group screening (14, 47). However, this may also have been affected by the timing of CeD diagnosis in relation to that of the AID. Both study groups demonstrated the female preponderance characteristic of CeD (2), although AIDs are in general more common in women (35), the sex distribution did not differ between the CeD + AID and CeD groups. Besides the already high overall proportion of women, this was partly due to the overrepresentation of men among subjects with T1D and IBD.

Contrary to the recommendations but in line with our previous experience (50–52), most of the participants lacked regular follow-up for CeD. Nevertheless, the CeD +AID and CeD groups had comparable GFD adherence and rate of serological and histological recovery. The fact that some patients were still TGA positive after 2 and even 4 years on a GFD and many had ongoing mucosal recovery is likely due to the often slow healing despite a strict diet (51, 52), although we cannot fully exlude ongoing gluten exposure. Earlier studies have focused mainly on patients with CeD and T1D and reported lower adherence figures than observed here (42, 53–56). Moreover, in contrast to our present and earlier findings (52, 57), lack of follow-up has predisposed to compliance problems (58, 59). The high prevalence and good general awareness of CeD in Finland may lessen the role of follow-up, which may not be the case in all countries (60). Furthermore, subjects recruited *via* the CeD society are likely more aware of the importance of a strict GFD. In fact, we recently also observed lower adherence in a special group of adult T1D patients diagnosed with CeD in childhood (8). Taken together, despite the good GFD adherence even among the non-followed-up patients here, we consider systematic long-term surveillance particularly important in subjects with concomitant AIDs.

Despite the equal adherence, CeD + AID patients reported more gastrointestinal symptoms and poorer general health than those with CeD only. It remains unclear if the symptoms were due to the active CeD or AID or to both, but it is important to realize that delayed diagnosis of CeD may predispose to suboptimal clinical response (26). Earlier research on psychological wellbeing in patients with CeD and concomitant other AID is scant and again concentrated on T1D. At least in adolescents there have been no major differences between CeD + T1D and CeD or T1D only patients (61–63), but the treatment mode of T1D may affect quality of life on GFD (62). Notably, our sub-analyses with T1D and some other AIDs were mostly in line with the overall results, excluding some logical differences in symptoms. Altogether, the phenotype and health impact of different AIDs, or even of the same AID between individuals, may vary from asymptomatic to life-threatening and also fluctuate over time (64, 65). This makes it extremely difficult to decide on disease-specific screening and make follow-up recommendations for CeD, which is also why we analyzed all the included AIDs together. The results obtained call for a more individualized follow-up strategy focusing specifically on treatment compliance and alleviation of symptoms and possible health concerns associated with CeD, as well as on special features and disease activity of the co-existing AID. Although primary care surveillance may be sufficient for patients with uncomplicated CeD and AID, a low threshold for referral to gastroenterologist/endocrinologist can be recommended in case of a rare AID or more complicated disease course(s).

The main strengths of our study were the large and well-defined cohort of CeD and AID patients and the use of validated and widely used follow-up questionnaires to elicit gastrointestinal symptoms and physiological wellbeing. Retrospective collection of medical data at diagnosis and lack of knowledge of the exact timing of the AID diagnoses were limitations. Furthermore, the CeD patients were not screened systematically for AIDs, e.g., with specific laboratory tests, and underdiagnosis of the often clinically heterogenous AIDs may have affected to the results. Additionally, a small proportion of the study surveys were incompletely filled, and dietary adherence was not evaluated by validated questionnaires. The follow-up time on GFD was also relatively short in some of the patients and we were not able to assess the effect of the healthcare site of follow-up on the long-term outcomes. Finally, there is a risk for selection bias in the study recruitment, as for instance symptomatic patients and with more severe AID might have been more eager to participate.

To conclude, co-existing AIDs were common in patients with CeD but were not significantly associated with either the disease presentation at diagnosis or most of the long-term treatment and health outcomes. Nevertheless, the increased prevalence of persistent gastrointestinal symptoms and poorer self-perceived health on GFD indicates a need for more personalized follow-up strategies.

## Data availability statement

The original contributions presented in the study are included in the article/Supplementary material, further inquiries can be directed to the corresponding author.

## Ethics statement

The studies involving human participants were reviewed and approved by Ethics Committee of Pirkanmaa Hospital District, Tampere University Hospital, Tampere, Finland. The patients/participants provided their written informed consent to participate in this study.

## Author contributions

RT, KKu, AE, LK, and HH designed the study, contributed to data analysis and interpretation, and drafting of the manuscript. Additionally NV, SK, and KKa critically reviewed the results and made a substantial additional contribution to the manuscript. All authors reviewed and approved the final version.

## Funding

The work was funded by the Academy of Finland, the Sigrid Juselius Foundation, the Foundation for Pediatric Research, the Päivikki and Sakari Sohlberg Foundation, the University Consortium of Seinäjoki, and the Competitive State Research Financing of the Expert Area of Tampere University Hospital. The funders had no role in the design or conduct of the study.

## Conflict of interest

The authors declare that the research was conducted in the absence of any commercial or financial relationships that could be construed as a potential conflict of interest.

## Publisher's note

All claims expressed in this article are solely those of the authors and do not necessarily represent those of their affiliated organizations, or those of the publisher, the editors and the reviewers. Any product that may be evaluated in this article, or claim that may be made by its manufacturer, is not guaranteed or endorsed by the publisher.
