# Supplementary material for: Association of concomitant autoimmunity with the disease features and long-term treatment and health outcomes in Celiac disease
Source: Front Med (Lausanne). 2022 Nov 16;9:1055135. doi: 10.3389/fmed.2022.1055135 (PMC9709120; doi:10.3389/fmed.2022.1055135)
Supplement: Supplementary file 1 [file Table_1.DOCX]

| **Supplementary Table.** Coexisting autoimmune diseases in 806 adult celiac disease (CeD) patients | | | | | | | | | |
| --- | --- | --- | --- | --- | --- | --- | --- | --- | --- |
|  |  | Cases | |  | Women |  | Age at CeD diagnosis, yr | |  |
| Autoimmune disease | n | | % | | % | Median | | Quartiles | |
| All | 185 | | 23.0 | | 77.8 | 42 | | 24, 50 | |
|  |  | |  | |  |  | |  | |
| Thyroidal disease | 103 | | 12.8 | | 89.3 | 42 | | 31, 52 | |
| Type 1 diabetes | 27 | | 3.3 | | 48.1 | 13 | | 6, 37 | |
| Rheumatoid arthritis | 25 | | 3.1 | | 80.0 | 42 | | 19, 51 | |
| Sjögren’s syndrome | 11 | | 1.4 | | 90.9 | 44 | | 35, 50 | |
| Psoriasis | 10 | | 1.2 | | 70.0 | 45 | | 39, 51 | |
| SLE | 5 | | 0.6 | | 100 | 36 | | 30, 50 | |
| Lichen planus | 4 | | 0.5 | | 50.0 | 53 | | 51, 57 | |
| Multiple sclerosis | 4 | | 0.5 | | 100 | 47 | | 38, 49 | |
| Crohn’s disease | 3 | | 0.4 | | 33.3 | 18 | | N/A | |
| Addison’s disease | 2 | | 0.2 | | 100 | 34 | | N/A | |
| IgA nephropathy | 2 | | 0.2 | | 50.0 | 50 | | N/A | |
| Pemphigus | 2 | | 0.2 | | 100 | 42 | | N/A | |
| Ulcerative colitis | 2 | | 0.2 | | 0 | 10 | | N/A | |
| Other/single cases^1^ | 8 | | 1.1 | | 75.0 | 41 | | 22, 56 | |
| ^1^Alopecia totalis, ankylosing spondylitis, autoimmune enteropathy, autoimmune hepatitis, autoimmune neutropenia, chronic inflammatory demyelinating polyneuropathy, myasthenia gravis, pernicious anemia, vitiligo. SLE, systemic lupus erythematosus | | | | | | | | | |
